# Depths and limits of spontaneous categorization in a family dog

**DOI:** 10.1038/s41598-020-59965-6

**Published:** 2020-02-20

**Authors:** Claudia Fugazza, Ádám Miklósi

**Affiliations:** 1 Department of Ethology, Eötvös Loránd University, Budapest, Hungary; 2 MTA-ELTE Comparative Ethology Research Group, Budapest, Hungary

**Keywords:** Evolution, Psychology

## Abstract

Categorization has been tested in non-human animals after extensive training procedures under laboratory conditions and it is assumed that in non-primate species categorization relies on perceptual similarity. We report evidence of the ability to categorize objects in absence of specific training in a family dog with vocabulary knowledge of multiple toys, including exemplars of 4 categories. Our experimental design was devised to test categorization in absence of specific training and based on the spontaneously learned vocal labels of the categories, a condition that mirrors human studies more than previous experiments on non-human animals. We also observed that the dog’s categorization skills were more accurate when, prior to the categorization test, she was given the opportunity to play with the novel exemplars, suggesting that category representations arise not only from physical resemblance, but also from objects’ affordances (function).

## Introduction

Categorization is the mental ability to group discriminable objects or events into classes by means of some principle or rule and to respond to them in terms of their class membership instead of as individual stimuli (e.g.^1,2^).

Categorization has been in the focus of research for both human (e.g.^3^) and non-human animals (e.g.^4,5^). In humans, according to most researchers, categories underpin language learning in that they allow labels to be attached to categories of items^6^. For instance, it has been shown that naming different objects with the same name highlights commonalities among them and promotes categorization, even in infants as young as 6 months^7^.

Some linguistic competence makes it simpler to test categorization skills (i.e. it becomes possible to “request” the subject to select an object from a verbally identified category, by using its name). However, studies on pre-verbal infants showed that language is not needed for categorization to emerge^2^. Thus, categorization skills are not necessarily restricted to humans, although it seems that language trained apes perform better than non-language trained subjects in complex categorization tasks^8^.

Since the seminal work of Herrnstein and Loveland^9^ showing that pigeons can discriminate pictures with people from pictures without people, categorization abilities have been studied in several additional non-human animal species. For example, birds (e.g.^10^) and also fishes^11^ have been found to categorize stimuli based on perceptual cues, while research regarding mammals has mainly focused on primates (for review see^4,12^).

A typical categorization experiment on non-human subjects involves two phases^1^: training with a set of exemplars from a given category until a predetermined performance is achieved, followed by^2^ a test with a mixture of novel exemplars from the same category and non-exemplars. The degree to which subjects correctly sort these test items into category members versus non-members is considered evidence for categorization performance. While the ability to categorize is assumed to be of obvious advantage in the wild, as it reduces the efforts needed to acquire an appropriate response to different stimuli belonging to the same category, most previous studies on categorization abilities were performed under artificial conditions (i.e., in the laboratory), after massive training of the subjects to reach a predetermined criterion (e.g., Bovet and Vauclair^13^ trained baboons on 1460 trials to criterion; D’Amato *et al*.^14^ trained cebus monkeys on more than 1000 trials to criterion; Range *et al*.^15^ trained dogs on 720 to 2040 trials to criterion before starting the categorization of items). Studies about spontaneous categorization skills, in absence of training and under natural (i.e., non-laboratory) conditions are lacking.

It is commonly assumed that, while humans take into account the function of objects, besides perceptual cues, to form mental categories (e.g.^16,17^), non-human animals tend to categorize based on the perceptual features of the stimuli^4^. However, non-human animals are tested after intensive training procedures, during which the rules to be followed for categorization are established by the experimenters, typically based on perceptual features. This may strongly determine the principles used by the animals to categorize novel exemplars during the tests. Moreover, training and testing in the case of non-human animals often involves the use of photographs of the exemplars^11^, therefore the possibility to learn about the objects’ affordances is lacking or very limited.

Little is known about the role of objects’ function in non-human animals’ categorization. Some categorization based on affordances has been shown to extend not only to humans, but also to non-human primates (4 for review). Baboons, for example, after massive training, were shown to categorize “food” and “non-food” items^13^, thereby challenging the assumption that this type of categorization is linked to language^18^. Furthermore, studies on tool-related cognition in New Caledonian crows (e.g.^19^) suggest that the ability to categorize based on object affordances might be more phylogenetically widespread than commonly thought. However, it is not known whether object affordances may guide categorization in non-primate species or whether their categorization skills are constrained only by perceptual similarity.

Despite dogs having gained importance as subjects of cognitive studies, relatively little research has been conducted on their categorization skills. After being trained to distinguish dog vocalizations from non-dog sounds, dogs were found to discriminate novel (i.e., not trained) sound stimuli belonging to dogs from sound stimuli not belonging to dogs^20^ and, after being trained on pictures of dogs and pictures of landscapes, dogs were observed to discriminate novel pictures belonging to these two categories^15^. Dogs were also recently shown to identify novel odors as members of a trained odor category^21^. These studies showed that dogs, after being specifically trained on a given set of stimuli, are able to categorize novel samples based on perceptual similarity (visual, auditory or odor). Chaser, a Border collie that had been extensively trained to learn over 1000 object-names, was also successfully trained to learn common nouns that refer to categories, including discriminating objects with which he was allowed to play from objects that he was not allowed to interact with^22^. Importantly, it should be considered that Chaser’s categorization skills were tested after the dog had been specifically trained to learn and generalize object categories until a predetermined performance was achieved. Thus it is not known whether and how dogs would spontaneously categorize, in absence of specific training.

Among the perceptual cues that humans use to categorize, color seems to play a major role and it has been suggested that categorization based on color might be an innate predisposition^23^. Color categorization has been found in several animal species as well (e.g., zebra finches^10^; fishes – e.g., triggerfish^11^; Chimpanzees^24^). Dogs have only two types of cone photoreceptors in their retina (those allowing perception of blue and yellow) therefore, they have a dichromatic color perception^25,26^. However, it has been claimed that dogs can also distinguish between red and green, at least under some brightness conditions^27^. It has been shown that dogs rely preferentially on color cues (blue and yellow), rather than brightness, in a two-way choice task^28^. However, little is known on whether dogs would spontaneously rely on color to categorize objects. We suggest that color is unlikely to be a fundamental, biologically relevant feature of objects for dogs (see also^29^) and, consequently, we would not expect them to spontaneously use color to categorize when other more relevant features can be used.

To investigate spontaneous categorization skills, we tested a family dog named Whisky, a 4-year old female Border collie with extensive vocabulary knowledge of multiple toy names spontaneously acquired during unplanned playful interactions with her owners. Among her named toys, Whisky also had some exemplars of toys belonging to one of 4 categories (balls, ropes, rings and Frisbees) and toys of different colors (blue, yellow, red, green, orange). The aim of this study was to test whether, despite the lack of specific training, the dog would be able to categorize novel exemplars of objects belonging to those categories, since previous studies on categorization typically relied on extensive training of the subjects in laboratory conditions. This allowed us to test whether non-human animals may spontaneously form mental categories, when exposed to their exemplars in a natural context.

Additionally, we aimed to explore whether increased attention to the objects and experience with their affordances, beside perceptual features (shape), play a role in the dog’s spontaneous categorization tendency. In order to achieve this, the subject was exposed to the novel toys in two conditions, before testing her ability to categorize novel exemplars belonging to those categories: (1) Exposing her to a brief play session with the toys before the categorization test (*Play condition*) and (2) Without this previous play interaction, but only allowing the dog to explore the toys before the test (*Exploration only condition*). In every categorization test of every condition, a novel set of toys was used.

We hypothesized that the natural, unplanned exposure to the objects belonging to the different categories and to their verbal labels received during her life in a human family allowed the dog to spontaneously form some mental categories of those objects. We also wanted to gain some insight on whether a brief experience of the typical type of play with the toys belonging to different categories – i.e., the way one plays with balls or ropes etc. – and the consequent increased attention to the objects, would enhance the dog’s accuracy in categorizing the novel exemplars. Additionally, we tested whether the dog had formed some color category by testing her ability to select familiar toys based on their color.

## Results

### Baseline: retrieving known toys based on their verbal labels (“names”)

We first tested Whisky’s knowledge of the proper name of all the toys available at her house, to which her owners had given a name (59 available toys, including also the toys of the 4 categories) by asking Whisky to fetch all of them, one by one, upon hearing their names.

**Figure 1:**
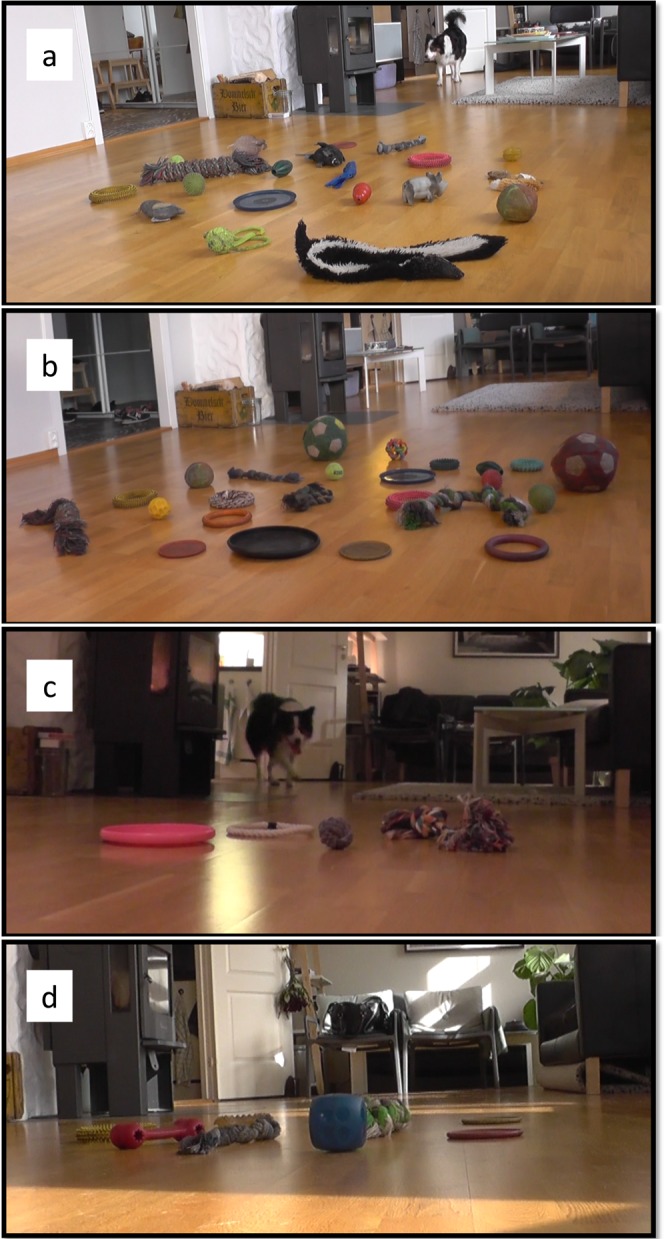
Experimental setup in the different tests. In the baseline (**a**), 20 randomly chosen familiar objects (including those belonging to the different categories) are on the floor; In the Test with familiar objects of the familiar categories (**b**), all the 25 objects belonging to the 4 categories (balls, Frisbees, rings, ropes) are on the floor; In the categorization test (**c**), 4 novel toys belonging to the 4 categories are on the floor. In the color categorization test (**d**), 8 objects of 5 different colors are on the floor.

Before the test started, 20 randomly chosen toys were laid on the floor of the living room by the experimenter – out of view from the owner who stayed in the kitchen for the whole duration of the tests. During the test, the owner requested the toys in a predetermined randomized order by saying: “Bring <object name>!”. Both the experimenter and the owner stayed in the kitchen, out of view of the toys during the test trials, thus they could not give any cue to guide the dog to a particular toy. Before a request to bring a named toy was made, the dog was free to move in the kitchen but was not allowed to approach the living room (the owner could encourage the dog to stay there by using food rewards). When a request was uttered, the dog went to the living room, selected a toy and brought it back to the owner, leaving it at his feet. We considered a choice to have been made by the dog when it appeared with a toy in its mouth. After every 5 trials, the experimenter took to the living room another set of 5 randomly chosen toys, so that the number of toys the dog could choose from always ranged from 16 to 20 (Fig. 1a).

Whisky was able to select the correct toy in 54 out of 59 trials (91.53% trials; Binomial probability P < 0.001).

### Test with familiar objects of the familiar categories

Within her set of named toys, Whisky had N = 10 different balls, N = 4 different ropes, N = 7 different rings and N = 4 different Frisbees. All of them had a name that was different from the others. The name of the toys belonging to the 4 different categories consisted always of an adjective or brief description and the word “ball”, “Frisbee”, “rope” or “ring”. The adjective or brief description of the compound name was always the first word of the compound name (e.g., “the small Frisbee”; “the colorful rope”). These toys were already included in the baseline test, together with the others in a random mode. Here we tested Whisky’s ability to recognize them based on their names, when all of the toys belonging to the 4 categories (and only those) were on floor for the dog to choose from (Fig. 1b). Therefore, in this test, 25 objects were on the floor and we carried out 25 trials, one per toy. The test procedure was almost identical to the one described for the baseline test and only differed in the type of toys available to choose from, belonging to the four test categories (i.e. all the familiar balls, Frisbees, ropes and rings). Additionally, the number of toys present per trial also varied from the baseline test (i.e., now 21–25 toys).

Whisky was able to select the correct toy in 18 out of 25 trials (72% trials; Binomial probability P < 0.001).

### Categorization tests

**Figure 2:**
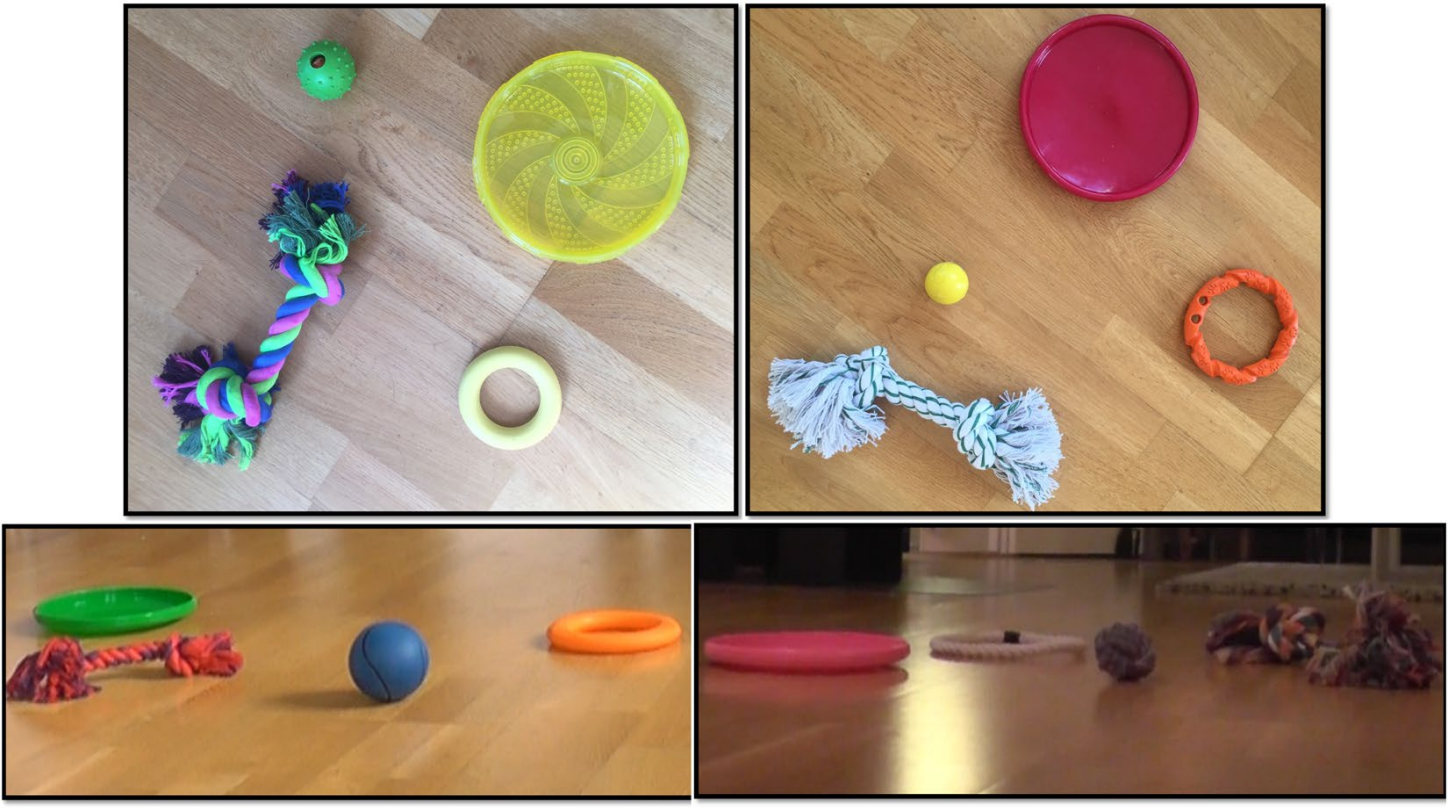
Samples of sets of toys used for the categorization test. In every test a novel set of 4 objects was used. The 4 objects belonged to 4 different categories: balls, Frisbees, ropes and rings.

To test whether Whisky had spontaneously formed some categories of the toys of which she had different exemplars, we tested her on novel exemplars of those 4 categories (i.e., “balls”, “Frisbees”, “rings” and “ropes”). In the test trials she was requested to choose e.g. “a rope” among a set of completely novel objects including an exemplar for each category (a rope, a ball, a Frisbee and a ring, Fig. 1c). All the exemplars of a given set were requested in a test in different trials and the order of the category to be requested in the trials was randomized. These novel toys belonged to the given category based on their shape but differed from the familiar named ones in some other features. We chose the toys from commercially available ones (Fig. 2). As Whisky was not trained specifically for categorization but was only exposed to unplanned playful interactions with the owners, her familiar toys varied in unplanned ways in size, color and material, which made it impossible to vary in a systematic way those features in the novel toys used during the tests. We chose the novel toys based on them differing from the familiar ones as much as possible and in at least two features (size and color and when possible also material – plastic, gum, cloth).

#### Exposure to the toys before the tests

To gain some insight into whether Whisky categorized the objects only based on their perceptual similarity or took into account their affordances as well, before every categorization test we exposed her to the novel toys in two conditions:

#### Play condition

Before testing, the dog was exposed to a brief (1 min) play session with her owner with 4 novel toys belonging to the 4 categories, in a random order, without naming the toys. The owner was asked to play in the typical way he would play with that type of toy. The owner only used short sentences which he typically used during playful interactions, such as “come here!”, “give it to me!” and “yes, good!” but never pronounced the name of the categories.

#### Exploration only condition

Whisky was only allowed to explore a novel set of 4 toys that were laid on the floor but did not play with them before the test.

After exposure to the toys in one condition, the categorization test was carried out as described above for the baseline test, but only the 4 novel exemplars, each of which belonging to a different category, were laid on the floor. As these toys were not given a proper name, the owner asked the dog to bring them by saying: “bring a <name of the category> ” (e.g., “bring *a rope*!”). After every trial, the toy that the dog chose was brought back to the living room, so that there were always all 4 toys for the dog to choose from. We carried out 8 categorization tests with 8 sets of 4 novel exemplars each, 4 categorization tests after exposure to the novel toys in the *Play condition* and 4 categorization tests after exposure to the novel toys in the *Exploration only condition*, in a randomized order (Table 1). The first two tests (one per condition) consisted of 8 trials each, 2 trials per object-category; the remaining 6 tests (three per condition) consisted of 12 trials each, 3 per object-category. In the first sessions we kept the number of trials to a minimum in order to ensure that the dog would not learn the name of the category (or of the given toy) during the test. We then slightly increased the number of trials to provide more accurate measurements of the dog’s performance, while still keeping the number of trials low enough to prevent learning during the test.

**Table 1:** Shows the experimental design of the categorization tests.

Exploration only Condition	Play Condition
Session 1 (8 trials)	Session 2 (8 trials)
Session 4 (12 trials)	Session 3 (12 trials)
Session 5 (12 trials)	Session 6 (12 trials)
Session 7 (12 trials)	Session 8 (12 trials)

The dog was tested in 8 categorization testing sessions, each of which on a novel set of toys.

Half of the tests were carried out after exposure to the toys in the Exploration only condition and the other half after exposure in the Play condition. The order of administration of the tests after exposure to the novel toys in the two conditions was randomized and the number of sessions indicated in the table (from 1 to 8) refers to the order with which the tests were performed.

In the categorization tests of the *Exploration only condition* Whisky retrieved the toy belonging to the correct category in 17 trials out of 44 (38.64% trials; One-tailed Binomial probability P = 0.027).

In the categorization tests of the *Play condition* Whisky retrieved the toy belonging to the correct category in 24 trials out of 44 (54.55% trials; One-tailed Binomial probability P < 0.001).

Although it is very unlikely that the dog learnt the names of the toys (i.e. that this particular toy was named e.g. “ring”) or their categories during the test, within such a small number of trials (2 trials per object in the first test session and 3 trials per object in the other test sessions), to fully exclude this possibility, we also calculated the results of the dog’s performance considering only the first trial for every novel object-category: in the categorization test of the *Exploration only condition* Whisky retrieved the toy belonging to the correct category in 7 trials out of 16 (43.75% trials; One-tailed Binomial probability P = 0.075); In the categorization tests of the *Play condition* Whisky retrieved the toy belonging to the correct category in 9 trials out of 16 (56.25% trials; One-tailed Binomial probability P = 0.005).

#### Effect of conditions

**Figure 3:**
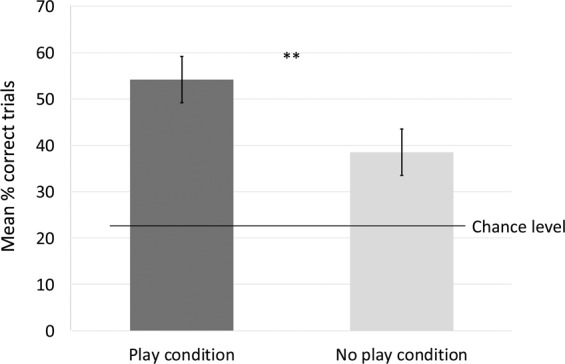
Mean percentage ± SD of correct trials in categorization tests after exposure in the *Play* and *Exploration only condition*. **Indicate significant difference between the performance in the two conditions (P < 0.05). The dog’s performance was significantly above chance in both conditions.

The percentage of correct trials per sessions differed significantly in the two conditions, with the dogs’ classification performance being more accurate in the *Play condition*: Wilcoxon-Mann-Whitney test: P = 0.014; (Fig. 3).

In the *Play condition*, out of 11 trials per given category, Whisky selected correctly the Frisbees in 8 trials (One-tailed Binomial Probability P < 0.001), the balls in 6 trials (One-tailed Binomial Probability P = 0.028), the ropes in 7 trials (One-tailed Binomial Probability P = 0.004) and the rings in 4 trials (One-tailed Binomial Probability P = 0.300).

In the *Exploration only condition*, out of 11 trials per given category, Whisky selected correctly the Frisbees in 4 trials (One-tailed Binomial Probability P = 0.300), the balls on 6 trials (One-tailed Binomial Probability P = 0.028), the ropes on 5 trials (One-tailed Binomial Probability P = 0.111) and the rings in 2 trials (One-tailed Binomial Probability P = 0.430).

As overall Whisky made many mistakes when requested to choose a ring, we calculated whether there was a specific alternative category that she chose when requested for a ring. Out of 22 trials in which rings were requested (and out of the 16 in which she made mistakes on the rings), in 5 trials she chose the ball, in 7 trials she chose the Frisbee and in 4 trials she chose the rope. None of them was above chance (Binomial Probability: P > 0.300).

### Color categorization test

Among Whisky’s familiar toys, there were also 15 objects of 5 different colors (3 blue objects, 3 yellow objects, 4 green objects, 3 red objects and 2 orange objects) that contained the word indicating their color in their names. The color was always the first word of the compound name. To test if Whisky had formed some color category, we tested her with a random subset of 8 familiar objects that had 5 different colors and that also contained the word indicating the color in their names - e.g. “blue dice” (Fig. 1d). The subset contained 2 red objects, 2 blue objects, 2 yellow objects, 1 green object and 1 orange object. The test procedure was identical to the previously described one, but the owner asked the dog to bring <name of color> (e.g. “Bring yellow!”).

The test consisted of 15 trials, 3 per color category, but was interrupted after 13 trials because the dog showed signs of stress (i.e., whining, panting and barking intensely).

Whisky retrieved the toy belonging to the correct color category in 3 trials out of 13 (23.07% trials; One-tailed Binomial probability P = 0.472). In two of those correct trials she brought a blue object and in one of them a yellow one. The blue and the yellow objects were requested 2 times in the 13 trials, thus Whisky correctly choose a blue object 100% of the times and the yellow one 50% of the times when these colors were requested.

## Discussion

We found the ability to categorize objects in absence of formal training in a dog with knowledge of multiple object names. The dog was able to select novel objects based on their categories upon hearing the verbal label of the category. Importantly, the innovative feature of this study is that the experimental design mirrors human studies more closely than typical animal studies due to two factors: the lack of specific training for categorization prior to the tests and the use of vocal labels to indicate the categories. While the study of categorization in non-human animals typically involves extensive training carried on until the subjects reach a predetermined criterion, our study subject did not receive any formal training aimed at teaching object categories. Compared to laboratory animals, Whisky’s experience is more similar to the experience of human infants living in a family and being naturally exposed to objects, their names and their categories. This allowed us to investigate the mental categories spontaneously formed by the dog during her daily life in a human family and to shed some light on the different features (or combinations of those) used to spontaneously categorize objects.

Whisky seemed not only to rely on perceptual similarity, but also on objects’ affordances, in order to assign novel items to a given category. Furthermore, she performed better when she was given the possibility to play with her owner with the objects, suggesting that increased attention to the objects and to their affordances (i.e., the way to play with them) enhanced her accuracy in the categorization task. Human infants are known to categorize on the basis of correlations between static and dynamic features of the objects, such as action, motion and function^17,30^ and this type of conceptual categorization is thought to be more generalizable and more abstract because it can survive changes in other perceptual features. The physical experiences with the novel toys in the *Play condition* significantly enhanced Whisky’s accuracy in the subsequent categorization task, as compared to when she was only allowed to explore (but not play with) the novel toys. These results suggest that object affordances (or a combination of affordances and perceptual cues – e.g., shape) also play a role in the categorization tendencies of species that are more phylogenetically distant from humans than primates^13^.

During the play sessions the owner was asked to play how he would typically play with that type of toy (e.g. the Frisbee was thrown in the air, while with the ropes he played tug of war). The possibility to experience familiar activities with novel toys is likely to have aided the dog in assigning them to the correct category more accurately then when she was only able to observe them.

However, other factors may also have played a role. During play with the novel exemplars, ostensive communication with the owner may also focus the attention of the dog to the object, which, consequently, may have facilitated categorization. Therefore, enhanced attention to the objects during the pre-test exposure may have also played a role in making the dog’s performance more accurate. Moreover, having the object in her mouth may add other perceptual dimensions that may support the categorization process. Another difference between the two conditions was that, in the *Play condition*, the level of activity was obviously more intense than in the *Exploration only condition*, as the dog chased and fetched the objects while playing with them with the owner. Therefore, we do not strictly exclude that these, or other possible differences between the two conditions contributed to enhance the dog’s performance. However, this study does illustrate the ability of a dog to categorize unfamiliar objects, even in the absence of function-related experience (at least for the “ball” category). We suggest that, in the case of the Play condition, it is likely that the enhanced attention given to different affordances of toys belonging to different categories was a very relevant and salient feature of the objects, that helped categorization more than their shape alone. As observed by Madole and Oakes^31^, the distinction between functional and perceptual categorization is not clear-cut. Instead, properties may vary along a continuum in terms of how much perceptual aspects (e.g., shape) and functional aspects (e.g. motion) determine them. For example, object affordance may present both functional and perceptual aspects and it could also be inferred by looking at the shape an object^32^. Therefore, in absence of further studies, we do not argue that this type of categorization is necessarily qualitatively different from categorization based on perceptual similarity.

Looking at the performance of the dog by isolating the objects of the different categories, we found that Whisky made most mistakes in both conditions when asked to select the rings. It is possible that she had not formed a “ring category” or that she did not recognize those particular objects as rings. It is also possible that, since the way the owner played with the rings was not very distinguishable from other categories (i.e., it contained elements of how he typically played with other toys, such as tug of war or throwing), the affordance of rings was not as clear cut as that of other categories. Whisky did not choose objects of a specific category instead of the rings; her choices were rather varied. She could correctly recognize all the other objects as belonging to their category in the *Play condition*, while her performance was above chance only for the category “ball” in the *Exploration only condition*. The lack of significance here may be only the effect of the small number of trials per given category. However, the dog’s better performance in the *Play condition* corroborates the role of the experience of playing with the toys and of experiencing their affordances in categorization.

The results obtained in the color categorization test suggest that Whisky did not rely on color when forming mental categories of objects. In this test she performed quite poorly and showed signs of stress, potentially due to not understanding the task. Her poor performance might be due to different factors but the small number of trials and the fact that it was not even possible to complete the test, do not allow to draw a firm conclusion. First of all, we suggest that object color is less biologically relevant for dogs^29^. Second, their dichromatic color perception may make colors less salient and distinguishable than some other features. Consequently, while it is known that dogs can discriminate colors^28^, this may not be the most relevant feature of objects they rely on, when spontaneously categorizing, at least when provided other more relevant possibilities.

The question of whether vocal labels can shape the early perceptual categories formed by young infants has received considerable attention. Althaus and Westermann^33^ showed that 10-month-old infants that were familiarized with a series of morphed stimuli along a continuum that could be considered as either one or two categories, divided the stimuli into two categories only when half of them were paired with one label and half with another label. The researchers suggested that labels and visual perceptual information interact in infants’ category formation, with labels constructively shaping category formation in infants, already in the pre-verbal age. Whether, as it has been proposed for humans (e.g.^18^), language and categorization abilities are also somehow cognitively connected deserves to be more deeply investigated in other species.

Since this study was conducted on one subject, the results should not be automatically extended to the dog population in general. However, these results do highlight that spontaneous categorization in absence of specific training is not a uniquely human feature. The extreme rarity of dogs with vocabulary knowledge^34^ makes it impossible to extend these results to a larger sample. Therefore, while we recommend caution in generalizing these results to the dogs’ population, we consider this study an important contribution to fill the gap in our knowledge of spontaneous categorization in non-verbal species. Hopefully this will stimulate researchers to devise methods to test categorization in absence of training in dogs and other species without knowledge of multiple object labels.

## Material and Methods

### Subject

We tested a 4-year-old female border collie named Whisky that lived as a family dog in a flat with her two owners. The owners reported that Whisky had learned the name of multiple toys through the years and they provided a list of the toys that were currently available at their house to be used for testing. The owners reported that, to attribute a name to a toy, they would pronounce it several times in a playful context with the dog, allowing the dog to take it in her mouth, asking to fetch it, then playing again with it, while naming it again. This way, Whisky learned the name of 59 different objects, including rubber and plastic toys of various shapes, plush toys and animals of various materials. Each of those toys had been given a proper name (e.g. “rat”) by Whisky’s owners.

The tests were conducted at the house of the owners (Norway) and the owner spoke the names of the name of the objects in Norwegian.

### Testing procedure

#### Baseline

- The randomly chosen toys were laid by the experimenter on the floor of the living room at approximately 20 cm from one another.
- The owner and the dog stayed in the kitchen, out of view of the toys.
- The experimenter returned to the kitchen
- The owner asked the dog to bring one of the toys by saying “bring <name of the object>”.
- If the dog brought the correct toy, it was verbally praised and received a treat.
- If the dog brought the wrong toy, the choice was scored as incorrect and the dog was not praised.
- After ever five trials the owner replaced the previously selected toys in the living room with another 5 randomly selected toys.
- All the toys were requested once (59 trials).

#### Test with familiar objects of the familiar categories

The procedure of the test with familiar objects of the familiar categories was identical to the one described above but the test consisted of 25 trials, as there were 25 objects to choose from (balls, ropes, rings and Frisbees) and all of them were requested once.

#### Categorization test

Before carrying out the test in which the dog was requested to select a novel toy based on its category, she was briefly exposed to the novel toys in 2 different conditions.

1. Exposure to the novel toys:

- In the *Play condition*, before the test, the owner was requested to play with the dog with the four novel toys, in a random order, in the usual way he would play with those (e.g. how he usually played with Frisbees, how he usually played with ropes etc.). The play session lasted for 1 minute for each novel toy, then the categorization test was administered. The owner was not allowed to name the toys, nor to pronounce the name of the categories during the paly session. When playing with ropes, the owner would typically let the dog take the rope in her mouth and play tug of war with her. When playing with balls, the owner would typically let the ball roll on the floor or toss it and allow the dog to fetch it; When playing with Frisbees, the owner would typically throw the Frisbee in the air, let the dog grab it and then play tug of war with it; When playing with rings the owner would typically let the dog grab it and play tug of war, sometimes after having thrown it in the air.
- In the *Exploration only condition*, the dog was exposed to the toys that were laid on the floor and was allowed to explore them (i.e., sniff and mouth them) in presence of the passive owner, who did not play with the dog, for the same amount of time.

2) Categorization test

The test procedure was identical to the one described above but, in every trial, after the dog brought a toy to the owner, the experimenter took the toy back to the living room before the next trial started. Therefore, all the 4 exemplars of the categories were always present for the dog to choose from. The demonstration video of the methods is available in supplementary information.

### Data analysis

To calculate whether the dog was better than chance at categorizing the novel objects we used one-tailed Binomial tests. For the binomial probability calculations of correct choices in every test, we set chance level based on how many objects were laid on the floor, from which the dog could choose.

In the baseline test, we calculated chance level conservatively at 0.06, as if there were always 16 toys to choose from (while they ranged between 16 and 20). In the test with familiar objects of the familiar categories, we also calculated chance level conservatively at 0.05 as if there were always 21 toys to choose from (while they ranged between 21 and 25).

In the categorization tests the dog always chose from 4 toys, therefore we set chance level at 0.25.

In the color categorization test, the dog had 5 colors to choose from, therefore we set chance level at 0.2 (we note that this is rather conservative, because there were more exemplars of the same color).

### Ethical statement

All experiments were performed in accordance with relevant guidelines and regulations. The Institutional Committee of Eötvös Loránd University has approved the experiments of this study (N. PE/EA/691-5/2019).

### Owner consent

The owner of Whisky volunteered for this study and his consent was taken.

## Supplementary information

Supplementary Video

## Data Availability

The datasets generated during analyzed during the current study are available from the corresponding author on reasonable request.
